# The Neural Basis of Decision-Making and Reward Processing in Adults with Euthymic Bipolar Disorder or Attention-Deficit/Hyperactivity Disorder (ADHD)

**DOI:** 10.1371/journal.pone.0037306

**Published:** 2012-05-18

**Authors:** Agustin Ibanez, Marcelo Cetkovich, Agustin Petroni, Hugo Urquina, Sandra Baez, Maria Luz Gonzalez-Gadea, Juan Esteban Kamienkowski, Teresa Torralva, Fernando Torrente, Sergio Strejilevich, Julia Teitelbaum, Esteban Hurtado, Raphael Guex, Margherita Melloni, Alicia Lischinsky, Mariano Sigman, Facundo Manes

**Affiliations:** 1 Laboratory of Experimental Psychology and Neuroscience, Institute of Cognitive Neurology and Institute of Neuroscience, Favaloro University, Buenos Aires, Argentina; 2 National Scientific and Technical Research Council, Buenos Aires, Argentina; 3 Laboratory of Cognitive and Social Neuroscience, Universidad Diego Portales, Santiago, Chile; 4 Integrative Neuroscience Laboratory, Physics Department, University of Buenos Aires, Buenos Aires, Argentina; 5 Pontificia Universidad Católica de Chile, Santiago, Chile; 6 Pontificia Universidad Católica Argentina, Buenos Aires, Argentina; 7 Neurored, Universidad Surcolombiana, Neiva, Colombia; 8 Laboratory for Behavioral Neurology and Imaging of Cognition, University of Geneva, Geneva, Switzerland; CSIC-Univ Miguel Hernandez, Spain

## Abstract

**Background:**

Attention-deficit/hyperactivity disorder (ADHD) and bipolar disorder (BD) share DSM-IV criteria in adults and cause problems in decision-making. Nevertheless, no previous report has assessed a decision-making task that includes the examination of the neural correlates of reward and gambling in adults with ADHD and those with BD.

**Methodology/Principal Findings:**

We used the Iowa gambling task (IGT), a task of rational decision-making under risk (RDMUR) and a rapid-decision gambling task (RDGT) which elicits behavioral measures as well as event-related potentials (ERPs: fERN and P3) in connection to the motivational impact of events. We did not observe between-group differences for decision-making under risk or ambiguity (RDMUR and IGT); however, there were significant differences for the ERP-assessed RDGT. Compared to controls, the ADHD group showed a pattern of impaired learning by feedback (fERN) and insensitivity to reward magnitude (P3). This ERP pattern (fERN and P3) was associated with impulsivity, hyperactivity, executive function and working memory. Compared to controls, the BD group showed fERN- and P3-enhanced responses to reward magnitude regardless of valence. This ERP pattern (fERN and P3) was associated with mood and inhibitory control. Consistent with the ERP findings, an analysis of source location revealed reduced responses of the cingulate cortex to the valence and magnitude of rewards in patients with ADHD and BD.

**Conclusions/Significance:**

Our data suggest that neurophysiological (ERPs) paradigms such as the RDGT are well suited to assess subclinical decision-making processes in patients with ADHD and BD as well as for linking the cingulate cortex with action monitoring systems.

## Introduction

Decision-making is essential in our daily lives. We make many different decisions; some are based on risk and predictability, whereas others are based on uncertainty or emotional heuristics. Current research examining decision-making has assessed multiple processes engaged in this complex cognitive ability. Evidence from animals, healthy human volunteers and neuropsychiatric patients [1]–[7] highlight the role of the frontostriatal and limbic loops in this process. Despite some discrepancies between different decision-making models, three neural systems are thought to be involved in the frontostriatal and limbic loop: a stimulus encoding process (i.e., the orbitofrontal cortex), a reward-based action selection and monitoring system (i.e., the cingulate cortex) and expected reward processing (i.e., the basal ganglia and amygdala). Thus, impaired decision-making may be the result of different deficits in these (or other) brain areas and may be affected differentially by disparate scenarios. Consequently, the nature of these decision-making deficits is dependent upon context and disease.

Bipolar disorder (BD) and attention-deficit/hyperactivity disorder (ADHD) usually share clinical symptoms, present high rates of comorbidity and are challenging to differentiate from one another clinically [8]–[10]. These disorders affect people by presenting problems in common decision scenarios that have social and vocational effects. Decision-making impairments have been reported in patients with ADHD [11]–[13] and those with BD [14], [15]. Nevertheless, previous decision-making studies using neuropsychology methods have shown inconsistent results for both disorders. In addition, no previous report has assessed a decision-making task that includes the examination of the neural correlates of reward and gambling in adults with ADHD and those with BD. Finally, no research has yet compared these disorders with regard to decision-making domains.

We hypothesized that, given the distributed neural network involved in decision making, the comparisons among groups would disentangle the different processes with regard to separate subtasks. Moreover, if both disorders present impaired decision-making related to their specific symptomatology, decision making deficits may be associated with specific ADHD and BD clinical/neurocognitive profiles.

This study assesses decision-making using the behavioral and neural correlates of different tasks in both ADHD and BD participants. We included affective, risky and rapid-decision gambling paradigms in order to test different aspect of decision making in patients. Specifically, we used an affective decision making task, the Iowa gambling task (IGT) [16], a task of rational decision-making under risk (RDMUR) [17] and a rapid-decision gambling task (RDGT) that elicits neurophysiological processes involved in the evaluation of the motivational effect of events [18]. We recorded event-related potentials (ERPs) from participants as they performed the RDGT. The RDGT elicits a feedback error-related negativity (fERN) modulated by reward valence and a P3 sensitive to reward magnitude [18], [19]. We also estimated the neural sources of these components. Finally, to assess the relationship between decision-making tasks and individual differences, a correlation analysis of the clinical-neuropsychological participant profiles was performed.

## Materials and Methods

### Participants

Fifty participants (BD: n = 13; ADHD: n = 12; controls: n = 25) received a full clinical assessment and neurocognitive profile, and their ERPs were recorded. Patients in the BD and ADHD groups were selected from the outpatient population of the Institute of Cognitive Neurology using the following inclusion criteria: 1) aged between 18 and 54 years old; 2) diagnosed with Type II BD or adult ADHD according to the DSM-IV using the Structured Clinical Interview for DSM-IV (SCID); and 3) euthymia scores less than or equal to 8 points according to the Montgomery-Asberg Depression Rating Scale (MDRS) [20] and less than or equal to 6 according to the Young Mania Rating Scale (YMRS) [21] for at least 8 weeks and with no change in medication type or dosage over 4 months. Patients did not receive antipsychotics (only patients with mood stabilizers were included). Exclusion criteria were 1) other Axis-I diagnoses, except for generalized anxiety disorder and 2) a history of mental retardation, neurological disease, or any clinical condition that might affect cognitive performance. We assessed all participants using a standard diagnostic process that included neurological, neuropsychiatric and neuropsychological examinations. All patients with ADHD were taking methylphenidate, which was suspended on the day of the ERP recordings because this medication improves task performance [22]. Patients were excluded in the study if there was disagreement in diagnosis between the two independent raters.

We recruited 25 healthy controls matched for sex, age, handedness, and years of education from a larger pool of volunteers who did not have a history of drug abuse or a family history of neurodegenerative or psychiatric disorders.

### Ethics

All participants provided written informed consent in agreement with the Helsinki declaration. Although some of the participants have diagnosis of ADHD or bipolar conditions, any of those disorders implied a reduced capacity to consent. The Ethics Committee of the Institute of Cognitive Neurology approved this study.

### Clinical, symptomatic and neuropsychological assessment

All participants completed a series of psychiatric and behavioral questionnaires to establish a clinical symptom profile that included depression, mania, impulsivity, anxiety, attention and hyperactivity/impulsivity scores. The Beck Depression Inventory II [23] and the Montgomery-Asberg Depression Rating Scale [20] rated depression. The Young Mania Rating Scale [21] and the Barratt Impulsiveness Scale [24] rated mania and impulsivity, respectively. The State-Trait Anxiety Inventory [25] rated anxiety. We obtained an ADHD symptom profile from the inattention and hyperactivity/impulsivity scores of the ADHD Rating Scale for Adults [26].

A general neuropsychology test evaluated participants' basic attention and memory processes. Several tests, including the INECO Frontal Screening [27], evaluated executive functioning. Digit and symbol searching and forward digit span tasks [28] evaluated attention, visual scanning and the efficient production motor responses. The Rey Auditory Verbal Learning Test [29], which is composed of verbal learning, immediate and delayed recall and a distractor list, evaluated memory. Several tests evaluated executive functioning. The INECO Frontal Screening [27] assessed frontal lobe function via several subtasks. Trail Making B [30] assessed attentional flexibility and attentional speed. Backward digit span, letter-number sequencing and an arithmetic test [28] assessed mental manipulation and working memory. A go/no-go task that included correct, incorrect and omitted responses as percentages and reaction time assessed inhibitory control. We also included a phonological fluency task.

### Decision-making tasks

#### IGT

The computerized version of the IGT [1] involves continuous card selections from four separate decks (A, B, C and D) and is complete after 100 selections. Each card choice is awarded a certain number of points (equivalent to small monetary incentives), but some choices yield penalties. Card choices from Decks A and B (“high risk”) generate large wins ($100) but also heavy losses that may lead to an overall debt. Decks C and D (“low risk”) generate smaller wins ($50 per choice) but also smaller penalties. Persistent selections from these decks yield a profit. The dependent variable of this task is the net score, which is calculated by subtracting the number of choices from the high-risk decks (A+B) from the choices from the low-risk decks (C+D). To quantify the change in decision-making across the course of the task, we divided this task into 5 blocks, each with 20 consecutive card choices [16]. In addition, we compared participants' net score on the first (1+2) and last (4+5) blocks.

#### The RDMUR Task

We designed a simplified computer gambling task based on a modified version of blackjack [17], [31]. At the beginning of the task, participants read the following instructions on screen: “*In this deck, there are 10 cards. Nine cards are “good,” and one is “bad”. You will win one dollar for each good card you draw; however, if you draw the bad card, then you will lose everything, and the game will end. You will keep whatever money you win, so try to play as well as possible. Choose one card at a time by clicking on it.*” We also told participants that the Joker was the bad card and that it was randomly placed within the set of ten cards. Finally, we told participants that they would play only once but could stop at any time to collect their prize. The task ended when participants stopped or drew the bad card. Using a mouse, participants could either select a face down card or stop the game by clicking the “check-out” box. Participants were unaware that any of the first 8 choices led to wins; in other words, the bad card was always the ninth card [17]. Because the expected value in this task is the highest after turning five cards [31], rational decision makers should stop after turning the fifth card.

#### RDGT

The RDGT allowed us to evaluate the motivational impact of events and the guiding choices of behavior [18]. Participants viewed two squares, each one containing either the numeral 5 or 25 (possible alternatives). Participants chose a square by pressing the corresponding button on the keyboard. After this choice, each square turned either red or green. If the square turned green, then the amount indicated on the square was added to their total amount. If the square turned red, then the amount indicated was subtracted from the total. The square not chosen also turned red or green at the same time; thus, participants not only discovered their gain/loss but also discovered what they would have gained/lost. These positive and negative feedback were triggered to obtain ERP waveforms during the EEG recordings. Each experimental session was divided into 24 blocks of 32 trials, and cumulative monetary awards were provided at the end of each block.

### ERP recordings and source estimation

EEG signals were sampled at 500 Hz from a Biosemi 128-channel system. The data were bandpass filtered online (0.1 to 100 Hz) and offline (0.3 to 30 Hz) to remove unwanted frequency components. We set the default reference as the link mastoids for recording. Two bipolar derivations monitored vertical and horizontal ocular movements (EOG). The EEG data that occurred 100 ms prior to, and 800 ms after, the stimulus onset were removed. Furthermore, we removed all segments with eye-movement artifacts from analysis using an automatic (spatial ICA) and visual procedure. We averaged artifact-free EEG segments to obtain ERPs.

#### Source modeling

Rather than using a single dipole model (e.g., aCC), we estimated the cortical current density mapping of fERN/P3 using a distributed model of 10000 current dipoles. Finally, we reported the activation of the cingulate cortex (anterior, medial and posterior sections), the valence (wins minus losses) at fERN, and the magnitude (large minus small) at P3. Orientation and dipole locations were fit to the Montreal Neurological Institute's standard brain model. Next, they were adapted to the standard geometry of the EEG sensor net (BrainSuite software). All subsequent processing (i.e., source analysis and visualization) was obtained using BrainStorm software. An extension of the overlapping-spheres analytical model computed EEG forward modeling [32]. EEG data using dynamic statistical parametric maps (dSPM) estimated cortical current maps based on the weighted minimum-norm current estimate (wMNE) [33]. We computed an activation threshold from signal baseline.

We separately analyzed regions of interest (ROIs) at the anterior, medial and posterior cingulate cortex (aCC, mCC and pCC, respectively) in both hemispheres using a Tzourio-Mazoyer partition [34]. Evoked responses in each ROI (see below) were indexed as the absolute power of all current sources for each ROI. Next, we reported the mean values of the three ROIs for valence (wins minus losses) at the fERN latency and magnitude (large minus small) at the P3 latency.

### Statistical Analysis

An ANOVA and Tukey's HSD post-hoc comparisons (when appropriate) compared demographic, neuropsychological and reaction time data across all three groups. The chi square test (*X* ^2^) examined categorical variables (e.g., sex). For the RDGT, we averaged accuracy and the ERP amplitudes for wins and losses (valence factor) as well as for large and small values (magnitude factor). We included a between-subjects factor for the group (patients with BD, those with ADHD, and controls). Offline processing and EEG data analysis were performed using Matlab. After a valence and electrode position analysis of the fERN [18], [35], we selected the FCz site for all analyses based on the higher win-loss amplitude discrimination. A 225–281 ms timeframe for fERN and a 372–464 ms timeframe for P3 were selected for mean amplitude analysis. Although the P3 has more of a central distribution, its effects are reliable at FCz [19], [36].

To perform correlations between the ERPs and the neuropsychology tests, we calculated global scores for (a) valence (fERN: wins minus losses) and (b) magnitude (P3: large minus small). Spearman's rank examined these global scores with regard to all clinical and neuropsychological tests after correcting for multiple correlations comparisons (at p<0.05, using Tukey HSD test).

## Results

Table 1 shows the results from the demographic, clinical, and neuropsychological assessments.

**Table 1:** The demographic, clinical, neuropsychological and decision-making results.

		BD (n = 13)	ADHD (n = 12)	Control (n = 25)	BD vs. ADHD	BD vs. CTR	ADHD vs. CTR
**Demographics**							
	Age (years)	40.1 (9.4)	31.4 (11.0)	35.1(11.2)	N.S	N.S	N.S
	Gender (F∶M)	5∶8	1∶11	9∶16	N.S	N.S	N.S
	Education (years)	16.5 (3.2)	15.5 (3.8)	17.2 (2.5)	N.S	N.S	N.S
**Clinical Profile**	Barkley						
	Inattention	7.6 (7.3)	13.2 (4.8)	2.5 (3.2)	**P<0.05**	N.S	**P<0.001**
	Hyperactivity	7.1 (6.3)	12.8 (4.2)	3.5 (3.3)	**P<0.05**	N.S	**P<0.001**
	BDI- II	8.0 (7.0)	17.4 (13.0)	5.7 (6.8)	N.S	N.S	**P<0.01**
	MADRS	3.2 (3.4)	4.6 (7.1)	1.0 (1.9)	N.S	N.S	**P<0.05**
	YMRS	0.3 (0.8)	2.2 (4.3)	0.3 (1.0)	N.S	N.S	**P<0.04**
	STAI						
	State	23.7 (6.7)	31.3 (10.6)	15.5 (8.7)	N.S	**P<0.05**	**P<0.001**
	Trait	27.6 (6.1)	30.9 (5.5)	19.1 (6.4)	N.S	**P<0.001**	**P<0.001**
	BIS- 11	54.2 (22.3)	59.1 (24.7)	40.9 (12.8)	N.S	N.S	N.S
**Decision Making**	IGT net score	1526.5 (483.0)	1571.0 (635.9)	1847.1 (564.1)	N.S	N.S	N.S
	IGT blocks 1 and 2	−1.3 (7.9)	−1.0 (6.3)	0.65 (7.1)	N.S	N.S	N.S
	IGT blocks 4 and 5	1.0 (8.4)	2.7 (8.6)	4.3 (8.2)	N.S	**P<0.05**	N.S
	RDMUR Task	7.2 (1.0)	6.8 (1.1)	6.7 (1.1)	N.S	N.S	N.S
	RT RDMUR (ms)	133235.4 (29023.5)	135952.1 (67787.4)	159151.5 (77508.5)	N.S	N.S	N.S
**Neuropsychological Measures**	Digits Forward (WAIS)	6.7 (0.9)	6.6 (1.3)	6.8 (1.1)	N.S	N.S	N.S
	Digits and Symbols (WAIS)	57.6 (14.7)	61.1 (15.5)	60.0 (9.4)	N.S	N.S	N.S
	Symbols Searching (WAIS)	33.2 (7.1)	31.1 (11.1)	35.0 (7.4)	N.S	N.S	N.S
	RALVT						
	Immediate	52.3 (8.3)	49.7 (11.9)	54.4 (6.6)	N.S	N.S	N.S
	Distractor List	7.2 (2.7)	7.6 (3.3)	7.6 (2.1)	N.S	N.S	N.S
	Delayed Recall	11.5 (3.0)	11.2 (4.0)	12.1 (2.2)	N.S	N.S	N.S
	Recognition	14.3(1.2)	13.0 (2.0)	14.6 (1.0)	N.S	N.S	N.S
	IFS						
	Total Score	24.9 (3.4)	25.9 (3.2)	27.3 (2.5)	N.S	**P<0.05**	N.S
	Motor series	2.5 (1.2)	2.9 (0.2)	2.7 (0.5)	N.S	N.S	N.S
	Conflicting instructions	2.9 (0.2)	3.0 (0.0)	3.0 (0.0)	N.S	N.S	N.S
	Go- no go	2.9 (0.2)	2.9 (0.2)	3.0 (0.0)	N.S	N.S	N.S
	Backward digits span	4.2 (1.2)	4.2 (0.7)	4.8 (1.1)	N.S	N.S	N.S
	Verbal Working memory	1.8 (0.7)	1.7 (0.6)	2.0 (0.0)	N.S	N.S	N.S
	Spatial working memory	3.2 (1.1)	2.8 (0.9)	3.5 (0.1)	N.S	N.S	N.S
	Abstraction capacity	2.8 (0.1)	2.4 (0.1)	2.9 (0.2)	N.S	N.S	**P<0.01**
	Verbal inhibitory control	4.7 (1.5)	5.0 (1.0)	5.2 (0.8)	N.S	N.S	N.S
	Digits Backward (WAIS)	4.8 (1.1)	4.5 (1.1)	5.3 (1.2)	N.S	N.S	**P<0.05**
	TMT-B	81.3 (52.6)	70.9 (25.0)	68.6 (15.4)	N.S	N.S	N.S
	Go/no- go Task						
	Correct Responses (%)	89.2 (20.3)	97.7 (5.0)	100 (0)	N.S	**P<0.05**	N.S
	Commission errors (%)	7.6 (19.8)	4.1 (6.0)	0.37 (2.0)	N.S	N.S	N.S
	Omission errors (%)	9.2 (20.3)	4.5 (7.0)	00 (0.0)	N.S	**P<0.05**	N.S
	Reaction Time (ms)	392.2 (70.7)	342.0 (131.7)	396.5 (46.9)	N.S	N.S	N.S
	LNST	12.3 (2.9)	11.0 (2.9)	12.4 (2.2)	N.S	N.S	N.S
	Phonologic Fluency	19.0 (6.0)	17.1 (4.7)	22.4 (6.9)	N.S	N.S	**P<0.05**

Abbreviations. BDI-II: Beck Depression Inventory; MADRS: Montgomery-Asberg Depression Rating Scale; YMRS: Young Mania Rating Scale; BIS- 11: Barratt Impulsiveness Scale; IGT: Iowa Gambling Task; RDMUR: Rational decision-making under risk; WAIS: Wechsler Adult Intelligence Scale; RALVT: Rey Auditory Verbal Learning Test; IFS: INECO Frontal Screening; TMT-B: Trail Making B; and LNST: Letters and Numbers Task.

### Demographic data

We did not observe between-group differences with regard to age (F[2], [47] = 1.52, p = 0.23), sex (*X* ^2^ [2] = 0.00, p = 1.00) or education level (F[2], [47] = 1.60, p = 0.23).

### Clinical evaluation

There was an expected between-group significant difference for the ADHD-RS-Inattention subscale (F[2], [47] = 12.46, p<0.001) and the ADHD-RS-Hyperactivity/impulsivity subscale (F[2], [48] = 8.90, p<0.001). Post-hoc comparisons (Tukey's HSD test, MS = 24.74; df = 47.00) showed that participants with ADHD had significantly higher inattention and hyperactivity/impulsivity scores compared to those with BD (p = 0.02, p = 0.03, respectively) and controls (both p<0.001). We observed a between-group difference for BDI-II scores (F[2], [47] = 6.13, p<0.01). Post-hoc comparisons (Tukey's HSD test, MS = 77.21; df = 47.00) revealed higher levels of depression for participants with ADHD (p<0.005) compared to controls. In addition, we observed a between-group difference for MADRS scores (F[2], [47] = 3.12, p = 0.48). Post-hoc comparisons (Tukey's HSD test, MS = 16.30; df = 47.00) revealed more severe depressive symptoms for patients with ADHD (p = 0.04) compared to controls. The YMRS scores also showed significant between-group differences (F[2], [47] = 3.52, p = 0.03). Post-hoc comparisons (Tukey's HSD test, MS = 4.76; df = 47.00) revealed higher levels of manic symptoms for patients with ADHD (p = 0.04) compared to controls. ADHD scored higher than those with BD on measures of inattention and impulsivity. This is an expected result, given that BD patients were euthymic. We did not observe between-group differences for the BIS-11 scores (F[2], [47] = 2.67, p = 0.10). However, significant differences between groups for STAI- State subscale (F(2,47) = 14.11, p<0.001) and STAI- Trait subscale (F(2,47) = 16.96, p<0.001) were observed. State subscale posthoc comparisons (Tukey test, HSD, MS = 74.28; df = 47.00) showed that BD (p = 0.02) and ADHD (p<0.001) participants had significantly higher scores than control subjects. Also, post hoc comparisons (MS = 38.29; df = 47.00) showed higher scores for Trait subscale in BD (p<0.001) and ADHD (p<0.001) patients compared with the control group.

In brief, patients with ADHD had higher scores of inattention, hyperactivity/impulsivity and depression than controls. In addition, patients with ADHD and those with BD had higher levels of trait anxiety than controls.

### Neuropsychological evaluation

The global score on the IFS showed significant differences between groups (F(2, 47) = 3.56, p = 0.03). Posthoc comparisons (Tukey test, HSD, MS = 7.58; df = 47.00) evidenced lower performance for the BD group compared with controls (p = 0.05). On abstraction capacity IFS subscale, significant differences between groups were observed (F(2, 47) = 5.21, p = 0.01). Posthoc comparisons (Tukey test, HSD, MS = 0.29; df  = 47.00) showed lower performance in the ADHD group (p<0.01) compared with controls. On the Go-no go Task, accuracy on go trials (F(2, 47) = 3.38, p = 0.04) and omission responses percentage (F(2, 47) = 3.28, p = 0.05), yield between groups significant differences. Posthoc comparisons on accuracy (MS = 110.38; df  = 47.00) presented lower performance for the BD group compared with controls (p = 0.03). Also, post hoc comparisons (Tukey test, HSD, MS = 119.54; df = 47.00) showed that BD had significantly higher omission responses percentage than did control subjects (p = 0.04). No differences were observed on either the commission responses percentage (F(2, 47) = 2.17, p = 0.12) or the reaction time (F(2, 47) = 2.63, p = 0.08).

Regarding the other measures of executive functioning, the score on verbal Phonologic Fluency Task presented significant differences between groups (F(2, 47) = 3.86, p = 0.02). Posthoc comparisons showed lower performance for the ADHD group compared with controls (p<0.01). The score on the Backward Digit Span evidenced a trend towards lower performance for the ADHD group (F(2, 47) = 3.14, p = 0.05). In contrast, no differences were observed between groups on the TMT-B (F(2, 47) = 1.12, p = 0.34), or the Letters and Numbers task (F(2, 47) = 1.32, p = 0.28).

In brief, the global score of the executive-function (IFS) showed significant differences between groups. Specifically, patients with BD had lower go/no-go IFS subscale scores compared to controls, and ADHD patients had lower abstraction capacity IFS subscale scores than controls. Furthermore, we observed impairments in patients with ADHD with regard to executive control and working memory.

### Decision-making (IGT and RDMUR)

**Figure 1:**
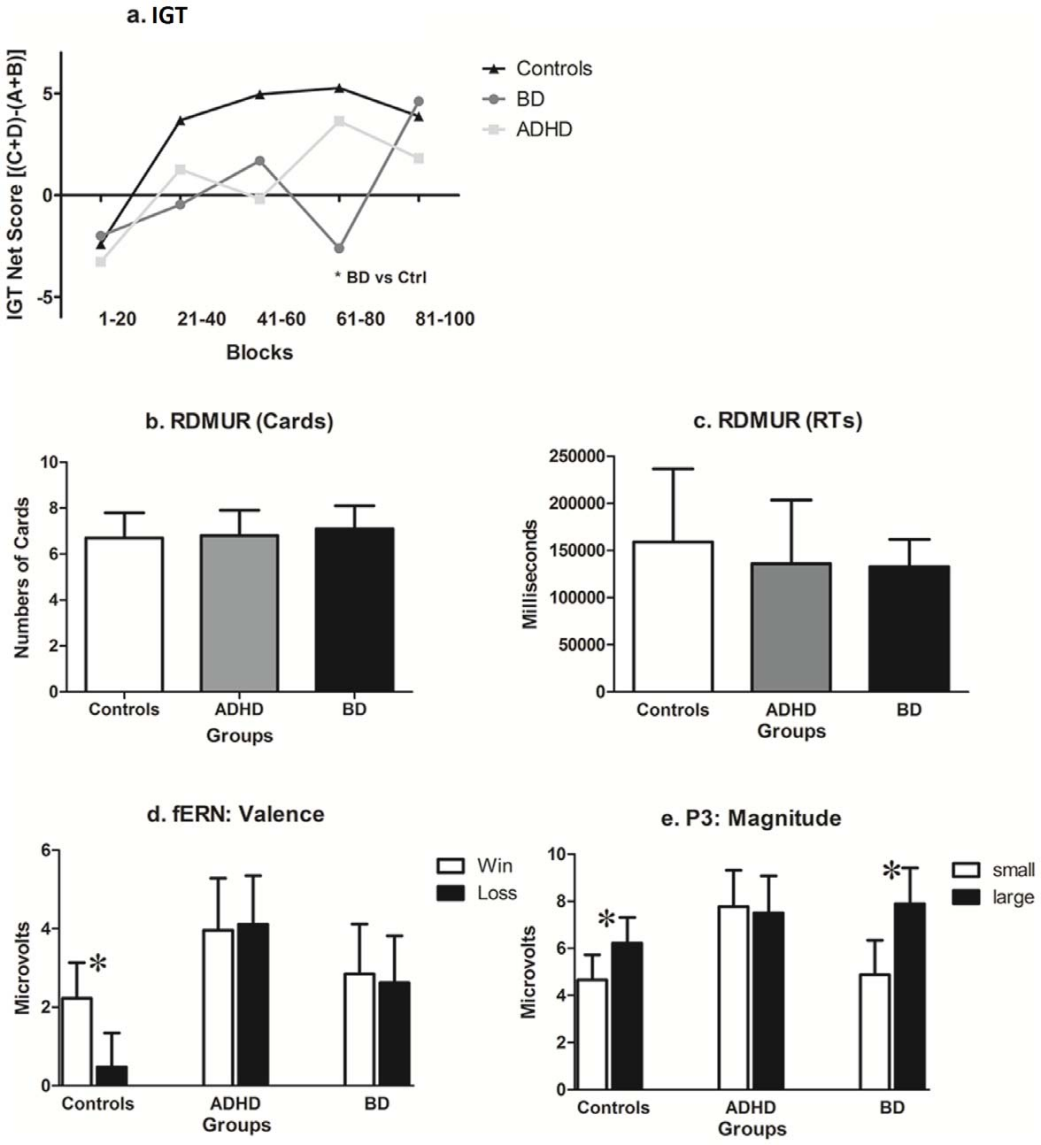
Decision-making task results (IGT, RDMUR and RDGT). A) IGT net score of Blocks 1 to 5; B) The number of cards selected in the RDMUR task; c) Total reaction time in the RDMUR task; D) Valence effects in the RDGT task; ERP mean amplitudes at the fERN timeframe; and E) Magnitude effects in the RDGT task; ERP mean amplitudes at the P3 timeframe. Boxes indicate SDs in b, c, d and e.

The IGT net score did not reveal a between-group difference (F[2], [47] = 1.37, p = 0.26). Furthermore, we did not observe an interaction between block and group. In order to look for more slight deficits, and to compare the initial and final blocks, we performed a separate analysis between the average of Blocks 1–2 and 3–4. Although an ANOVA did not find group differences in Blocks 1–2 (F[4, 90] = 1.02, p = 0.39), it did for Blocks 4–5 (F[4, 90] = 3.53, p = 0.01). Post-hoc comparisons (MS = 56.98, df = 47) revealed that patients with BD had impaired performances compared to controls (p = 0.01, see Figure 1.A).

When comparing RDMUR tasks (Figures 1.B and 1.C), we did not observe significant between-group differences with regard to total score (F[2], [47] = 0.44, p = 0.62) or total reaction time (F[2], [47] = 1.06, p = 0.34).

### The neurophysiological measures of Decision-Making

#### Reaction time

Regarding overall RTs, a group effect was obtained (F(2, 47) = 3.49, p<0.05). Post hoc comparison performed over this effect (Tukey HSD test, MSE = 1773, df = 47) evidenced that ADHD patients made longer responses (M = 1039 ms, SD = 122) than controls (M = 650 ms SD = 84). No differences were observed between BD (M = 816 ms, SD = 117) and controls. No main effects or interactions of valence and magnitude were observed in reaction times.

### RDGT: ERPs

#### fERN

We did not observe main effects of valence (F[1], [47] = 3.30, p = 0.07) or magnitude (F[1], [47] = 0.15, p = 0.69); however, as expected, we observed significant valence×group (F[2], [47] = 3.62, p = 0.03) and magnitude×group interactions (F[2], [47] = 5.11, p<0.005). To analyze the simple effects for control participants as well as those with ADHD and those with BD, we examined the fERN component of each group separately (see Table 2 for descriptive statistics).

**Table 2:** ERP descriptive statistics.

	BD mean (SD)	ADHD mean (SD)	Controls mean (SD)
**fERN**			
Valence			
Win	2.85 (1.27)	3.96 (1.32)	2.22 (0.91)
Loss	2.62 (1.20)	4.11(1.24)	0.48 (0.86)
Magnitude			
5	1.94 (1.26)	3.01 (1.31)	1.21 (0.90)
25	3.53 (1.20)	4.49 (1.25)	1.49 (0.86)
Interaction			
Win 5	1.82 (1.28)	4.75 (1.33)	1.90 (0.92)
Win 25	3.87 (1.36)	3.17 (1.42)	2.53 (0.98)
Loss 5	2.05 (1.30)	4.40 (1.36)	0.92 (0.94)
Loss 25	3.18 (1.19)	3.81 (1.24)	0.04 (0.86)
**P3**			
Valence			
Win	6.77 (1.44)	7.33 (1.50)	5.52 (1.04)
Loss	6.00 (1.55)	7.94 (1.62)	5.35 (1.12)
Magnitude			
5	4.21 (1.48)	7.77 (1.54)	4.65 (1.07)
25	8.89 (1.52)	7.50 (1.58)	6.22 (1.09)
Interaction			
Win 5	4.92 (1.47)	7.73 (1.53)	4.72 (1.06)
Win 25	8.62 (1.56)	6.94 (1.63)	6.33 (1.13)
Loss 5	4.82 (1.61)	7.82 (1.68)	4.59 (1.16)
Loss 25	7.17 (1.62)	8.06 (1.69)	6.12 (1.17)

Mean amplitude values of valence and magnitude for patients with BD, those with ADHD and controls.

Regarding Controls, we did not observe an effect of magnitude (F[1], [11] = 0.34, p = 0.85); however, as expected, a significant effect of valence (F[1], [11] = 10.69, p<0.01) revealed less positive amplitudes on trials with losses than those with wins. In addition, we observed a significant valence×magnitude interaction (F[1], [11] = 11.52, p<0.01). Post-hoc comparisons (MS = 2.27; df = 47.00) revealed that amplitudes after a large win were more positive than those after large (p<0.001) and small losses (p = 0.02).

Regarding patients with BD, we did not observe an effect of valence (F[1], [12] = 0.29, p = 0.59); however, we did find a significant effect of magnitude (F[1], [12] = 7.50, p = 0.01), revealing that the amplitudes associated with large reward were more positive than those associated with smaller ones. There was not a significant valence×magnitude interaction (F[1], [12] = 0.70, p = 0.41).

**Figure 2:**
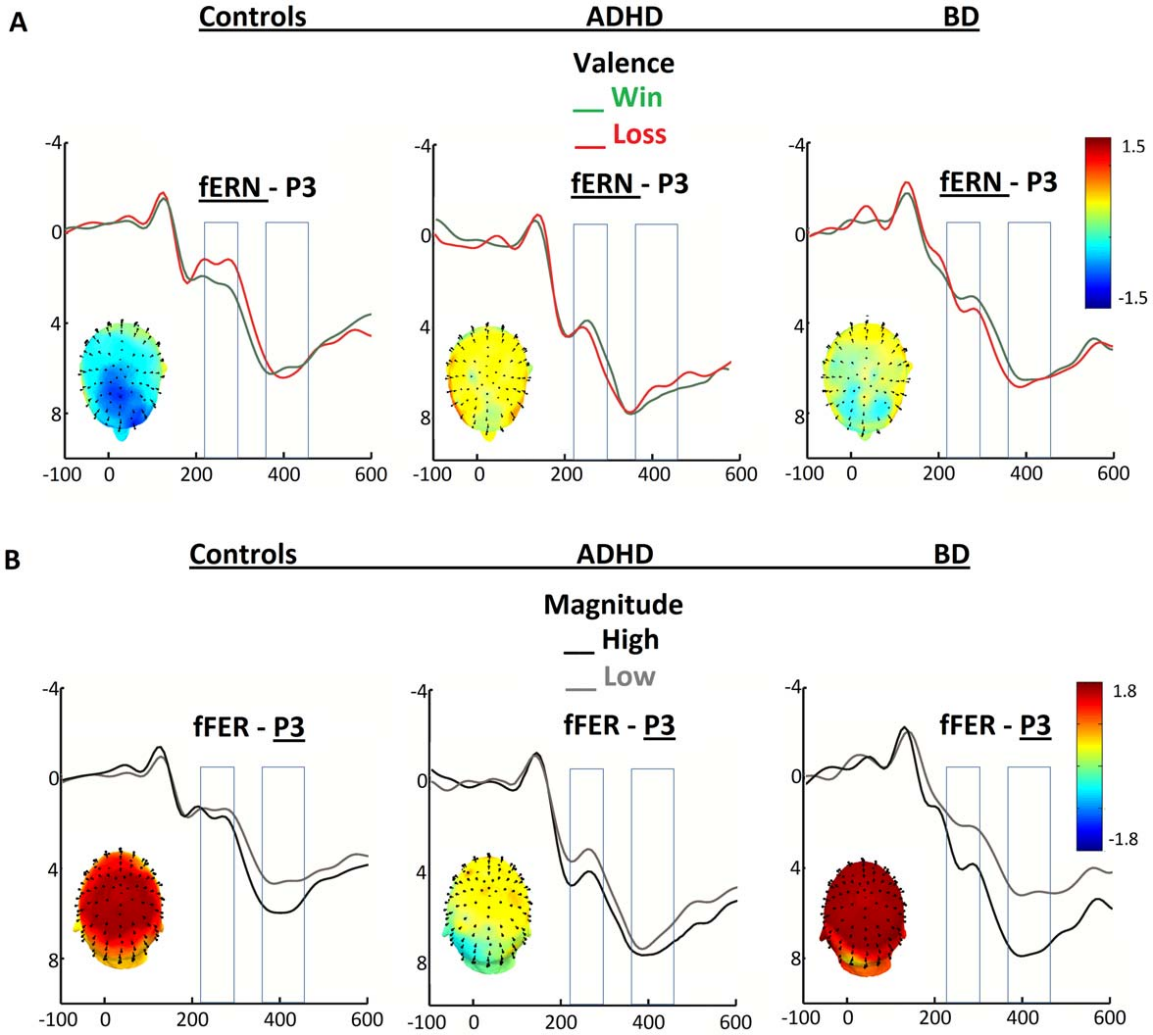
fERN and P3 modulation of valence and reward magnitude. A) FERN Valence modulation (wins vs. losses) in controls, patients with ADHD and those with BD.Voltage maps show the scalp modulations (losses minus wins) at the fERN timeframe. B) Magnitude modulation (large vs. small rewards) in controls, patients with ADHD and those with BD. The P3s of controls discriminated reward magnitudes whereas this effect was absent in patients with ADHD but enhanced in patients with BD. Voltage maps show the scalp modulations (large minus small) at the P3 timeframe.

Patients with ADHD did not presented a valence effect (F[1], [11] = 0.02, p = 0.87); nevertheless, an effect of magnitude (F[1], [11] = 3.54, p = 0.08) showed that, similar to patients with BD, the amplitudes associated with large magnitude were more positive than those associated with smaller ones. There was no significant valence×magnitude interaction (F[1], [11] = 1.12, p = 0.31). Figure 2.A shows the main effects of valence on fERN for all groups.

#### P3

There was a main effect of magnitude (F[1], [47] = 12.39, p<0.001). In addition, we observed a significant interaction between magnitude and group (F[2], [47] = 4.52, p = 0.01). As before, we analyzed the P3 component of each group separately.

The Control Group presented a significant effect of magnitude (F[1], [24] = 10.40, p<0.005) revealed that the amplitudes associated with large reward magnitudes were more positive than those associated with small magnitudes. There was not a significant effect of valence (F[1], [24] = 0.20, p = 0.65) or a valence×magnitude interaction (F[1], [24] = 0.14, p = 0.90).

Patients with BD also presented a significant effect of magnitude (F[1], [12] = 16.57, p<0.001) revealed that large reward magnitudes were more positive than small magnitudes. This effect was almost two times larger than the effect observed in the control group. There was not a significant effect of valence (F[1], [12] = 1.20, p = 0.29) or a magnitude×valence interaction (F[1], [12] = 1.35, p = 0.26).

Finally, patients with ADHD did not presented a significant main effects of magnitude (F[1], [11] = 0.10, p = 0.75) or valence (F[1], [11] = 0.28, p = 0.60) or their interaction (F[1], [11] = 0.32, p = 0.57).

Figure 2 shows the main effects of valence on fERN (2.A) and effects of magnitude (2.B) for all groups. Figures 1.D and 1.E summarize the ERPs results. Reward valence affected fERN in controls, but we did not observe an effect for either patient group. There were magnitude effects at P3 in the controls, which were reduced in patients with ADHD and enhanced in those with BD.

### Source activity

**Figure 3:**
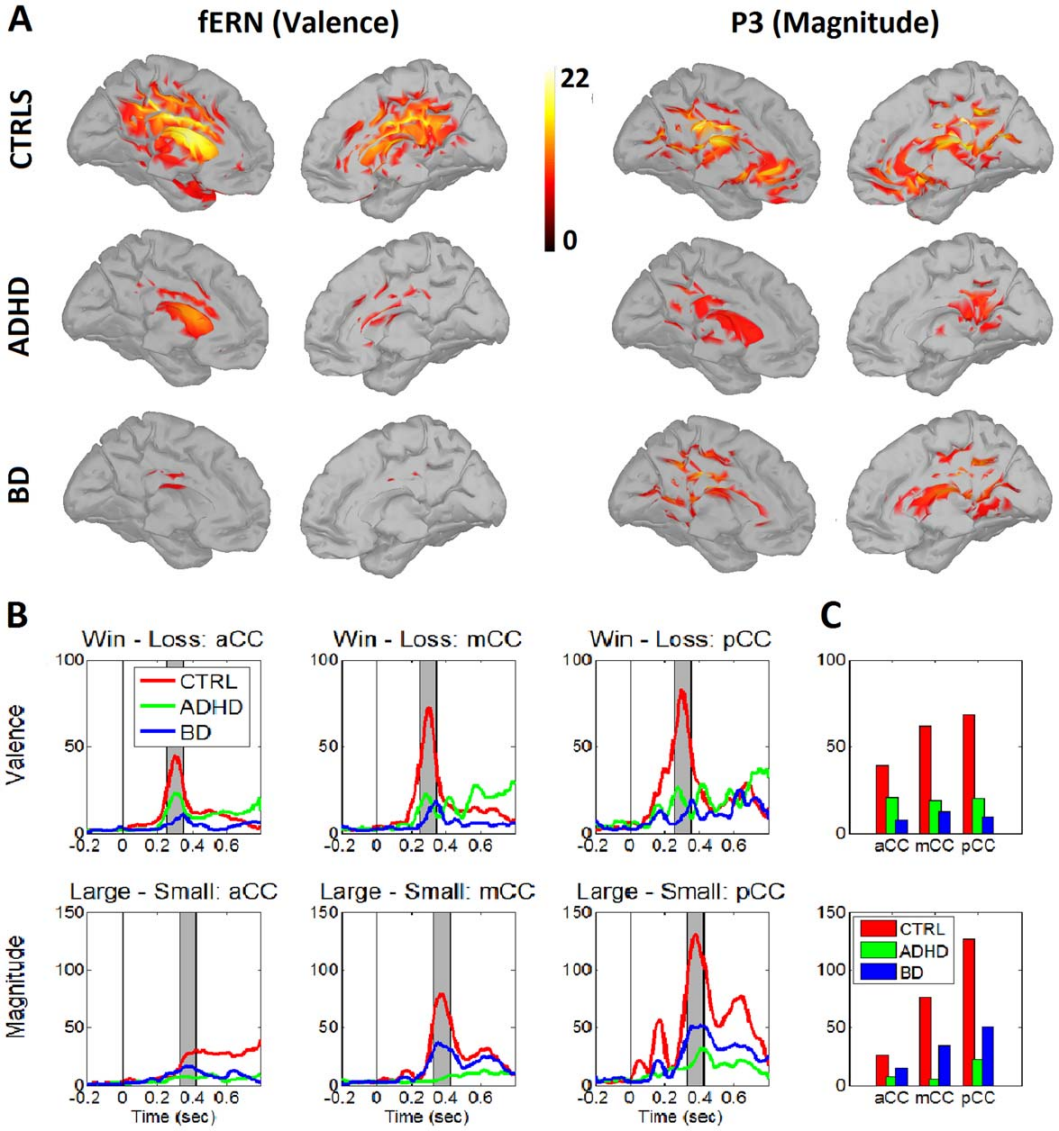
Cortical current density mapping of valence and reward magnitude. A. The source estimation of distributed valence dipoles (fERN, left) and magnitude effects (P3, right) for controls, patients with ADHD and those with BD. Color-map values represent the t-values of comparisons between signal and noise. B. A time-series of the absolute power activation evoked by valence and reward magnitudes at the anterior, medial and posterior cingulate cortex (aCC, mCC, pCC). C. The average values of absolute power at aCC, mCC and PCC for the valence and magnitude effects for all groups. We obtained the ROIs at aCC, mCC and pCC using a Tzourio-Mazoyer partition.

Figure 3.A shows the distributed activation evoked by the valence and magnitude of the rewards. Following a t-value comparison between signal and noise, valence presented a maximum over 268 ms (fERN) and magnitude presented a maximum over 432 ms (P3). Consistent with the ERP results, both patient groups presented a reduced activation of reward valence at fERN window compared to controls. The magnitude discrimination at P3 was more reduced in patients ADHD, followed by those with BD and controls. The source of fERN/P3 neural activity was estimated to be at different portions of the cingulate cortex (aCC, mCC and pCC). The cingulate activity at the fERN window (Figures 3.B and C, top) was reduced for patients with ADHD and those with BD compared to controls (valence effect). Medial and posterior cingulate regions of interest (ROIs) showed magnitude effects at P3, decreasing from controls to patients with BD to those with ADHD (Figures 3.B and C, bottom).

### Correlations (Clinical/Neuropsychological Assessments and ERPs)

#### Control Group

Impulsivity (BIS-11: r = −0.32, p<0.05) and depression (MADRS: r = −0.48, p<0.01) were negatively correlated with fERN win/loss discrimination. ADHD-RS-Inattention subscale scores were positively correlated with P3 amplitudes of magnitude discrimination (r = 0.40, p<0.05). Working memory (backward digits span) was negatively correlated with P3 magnitude discrimination (r = −0.40, p<0.05).

#### Patients with BD

Depression level (MADRS total score, r = −0.48, p<0.01 and Beck-II, r = −0.40, p<0.05) was negatively correlated with win/loss discrimination. Anxiety scores (STAI- Trait) were positively correlated with P3 magnitudes discrimination (r = 0.61, p<0.01). Inhibitory control (incorrect responses on a go/no-go task) was positively correlated with fERN win/loss discrimination (r = 0.37, p<0.05) and with P3 magnitude discrimination (r = 0.42, p<0.05). Go/no-go reaction times were negatively correlated with fERN win/loss discrimination (r = −0.38, p<0.05).

#### Patients with ADHD

Significantly high ADHD-RS-Inattention (r = −0.55, p<0.01) and ADHD-RS- Hyperactivity-impulsivity subscale scores (r = −0.39, p<0.05) were negatively correlated with fERN win/loss discrimination. With regard to executive functions, the IFS total score was positively correlated with fERN win/loss discrimination (r = 0.43, p<0.05). Working memory (numbers and letters, r = 0.54, p<0.05; WAIS-working memory index, r = 0.41, p<0.05) and attention (WAIS-digit score; r = 0.58, p<0.01) were also positively correlated with fERN win/loss discrimination.

## Discussion

This study revealed that fERN and P3 markers were more sensitive than behavioral measures in showing that the decision-making brain process is impaired in both groups of patients. We did not observe group differences using the IGT (except for final block analysis in patients with BD) or the RDMUR task. Nevertheless, the RDGT showed an abnormal data pattern within the patient groups.

Patients with ADHD presented a neural pattern indicative of deficient valence (fERN) and reward magnitude learning (P3). This pattern was associated with clinical evaluations of impulsivity, hyperactivity and inattention as well as impairments in executive function and working memory. Our data are consistent with the clinical features of ADHD with regard to decision-making: If the learning of valence and reward magnitude from the environment is impaired, then information concerning which decisions are most important will be reduced. Thus, decisions will be based on impulsivity or will not have a learned strategy.

Patients with BD presented a pattern of cortical modulation based on the saliency of reward magnitudes regardless of learning via feedback. There was no fERN valence modulation; but reward magnitude affected this variable. The P3 presented enhanced reward magnitude discrimination. The ERP pattern of our data was associated with mood states and inhibitory control. Those results are consistent with the hypothesis that there is reduced sensitivity to emotional reward or punishment contexts in BD [37].

Finally, we found reduced activity in the cingulate cortex (aCC, mCC and pCC) in both patient groups compared to controls. This activity was especially reduced for patients with BD at the fERN (valence) and those with ADHD at the P3 (magnitude). These results suggest that one of the main circuits associated with decision-making, the so called “action selection-monitoring system”, is impaired at neural level, for both groups but at different stages.

To our knowledge, this study is the first to compare the decision-making profiles of adults with ADHD and those with BD. Our results are novel in numerous aspects. First, we found that the behavioral measures of affective and risky gambling tasks are not sensitive enough in small samples to assess these disorders' well-known deficits in decision-making (but see limitations section regarding sample size). Second, we found that both patient groups show an abnormal neural processing of valence and reward magnitudes but that this pattern was associated with different clinical and neuropsychological profiles. Finally, our data are consistent with the models of cingulate cortex activation to reward, action selection and action monitoring.

### FERN, P3 and individual differences

The data of healthy volunteers confirms previous findings of fERN valence modulation [9], [19] and P3 magnitude modulation [37]. Our data are also consistent with the reports that suggest that decision-making processes, rewards and fERN/P3 sources are related to the aCC/pCC [38]–[42]. Nevertheless, no previous report has identified these effects in patients with ADHD or BD.

Higher levels of inattention, hyperactivity and impulsivity were associated with reduced fERN win/loss discrimination in patients with ADHD, which confirms the association between impulsivity and decision-making in these patients [43]. In addition, we found that executive function was associated to decision-making-related ERPs, which confirms previous reports of an association between inhibitory control, decision making and working memory. Difficulties in sustaining attention when updating working memory affect the decision-making of patients with ADHD. Likewise, our data are consistent with reports of reduced responses to rewards and reinforcements in children with ADHD [44], which suggests an impaired sensitivity to learning via feedback [12].

We found that anxiety and mood levels were correlated with reduced P3 magnitude discriminations in patients with BD. Previous studies have revealed a relationship between poor decision-making and anxiety and mood [6], [44], [45] as well as inhibitory control [14]. Consistently, our results showed that inhibitory control was negatively correlated with fERN win/loss discrimination and P3 magnitude discrimination in patients with BD.

Our multivariate analysis shows that psychopathological measures (e.g., inhibition, anxiety, impulsivity and depression) and executive functions can affect the decision-making processes. This is consistent with the current literature that highlights the involvement of executive functions [2], [46], [47] and social cognition [48], [49] in decision-making.

### The neuropsychological assessment of decision-making tasks in patients with ADHD and those with BD

Previous IGT studies testing patients with euthymic BD reported normal decision-making abilities. Recently, our group [50] and other laboratories [50]–[53] showed similar results. Nevertheless, two studies have found that patients with BD show poor decision-making performances [14], [15]. Methodological factors such as not including a control group [14], [15] or not excluding manic symptoms [14], [15] might account for those conflicting results. In addition, the absent behavioral deficits reported here would be related to the small sample size (see limitation section).

Likewise, the results of the IGT studies that test adults with ADHD are in conflict. Mantyla et al [13] found that although patients with ADHD have impaired performances on the IGT, they showed similar patterns of gambling to healthy controls. Furthermore, formal education mediated these group differences. Malloy-Diniz et al. [43] also found between-group IGT differences, but some participants with ADHD had histories of drug abuse or met generalized anxiety disorder diagnosis criteria. These controversial results would be partially explained by a lack of more comprehensive evaluation of decision making, as well as the influence of personality and state mood on IGT [54]. Ernst et al. [44] did not find differences between adults with ADHD and healthy controls. However, the limbic areas of patients with ADHD failed to activate. Similar to Ernst et al. [44] our results suggest that the subclinical neural processing of decision-making is impaired despite a lack of behavioral evidence.

The assessment of rational decision-making using ‘risky’ tasks is scarce in ADHD or those with BD. No reports have examined the former group, and previous results have found both impaired [49] and functioning [55] in patients with euthymic BD. We found no risky behavior (as measured by the RDMUR task) in either patient group. Confirming previous reports [56], the brain-behavior correlations evidenced that neural markers of decision making deficits might be related to behavioral impulsivity.

Finally, we found a neural pattern associated with reward magnitude and valence consistent with models of action selection monitoring [3], [4], [7], [57]. Reduced brain volume and abnormal activity in areas related to decision-making, including the cingulate cortex, have been reported in patients with ADHD [4], [58], [59] and those with BD [4], [60]. Moreover, reports on decision-making in moral paradigms using patients with BD [61] and short- versus long-term rewards in patients with ADHD [11] have shown abnormal activity in the cingulate cortex. Our results on source estimation suggest that the cingulate cortex when monitoring decision-making is an important marker of specific subclinical impairment and co-segregation in patients with ADHD and those with BD.

### Clinical and neuropsychological assessment in patients with ADHD and those with BD

As expected, patients with ADHD had higher inattention and hyperactivity/impulsivity scores than controls. Patients with ADHD also had higher levels of depression, which is common in this clinical population [62].

As we previously reported [62], both patients groups presented executive domain difficulties. Specifically, patients with BD showed poorer performance on a go/no-go task [63]–[65] and had inhibitory control failures. Patients with ADHD showed deficits in phonologic fluency compared to the control group [66].

### Limitations

Mainly, and similar to previous studies, the number of patients was restricted, and therefore more subtle differences may have been missed due to a lack of statistical power, and multivariate comparisons require replication using a larger sample. However, the exclusion of patients with comorbidities and those receiving medications that might modify their electrophysiological responses accounts for the small size of our sample. Future research should explore the possible associations of the neural-level findings of decision-making with measures of social and vocational functioning as well as insight and compliance, which are clinical dimensions usually affected by both disorders. Possibly, low numbers of subjects may have impacted on the lack of differences found between groups on the IGT as well as the finding of no risky behavior in either patient group, something that was at some extent unexpected. For example, in one study of the IGT in ADHD [43], a sample size of 50 individuals with ADHD and 51 controls was utilized. Consequently, IGT would in fact, with a larger sample, be able to pick up the decision-making impairments. In addition, age-related changes have been reported on the IGT, and it is possible that the wide age range used in the present study may be introducing error variance into the results. Nevertheless, neural markers were more sensible to detect specific impairments in the affected groups with a relatively small sample. Future studies comparing both behavioral and neural correlates of decision making in a larger sample would asses this issue.

Finally, as all previous reports comparing ADHD and BD patients, potential confounding effects of medication were not completely ruled out. As with almost all previous studies, BD patients in the current study were taking medications (but we did not include participants on antipsychotics). Although ADHD participants suspended the medication the day of recordings, long term effects of stimulants may have persistent effects on brain function. Therefore, we cannot discount the influence of these drugs on cognitive function. Assessing the same decision making task and comparing those effects in drug-naive participants (in order to avoid the possible long term effects of medication) would be additional steps.

### The clinical and theoretical significance of our findings

Our results reveal a clinical association between neural substrates and the well-known impairments of decision-making in patients with ADHD and those with BD. Neurophysiology may be able to examine brain abnormalities undetected by classic neuropsychology. The present results expand our previous work on decision-making from frontal diseases with clear structural damage [3], [27], [48], [67]–[71] to psychiatric diseases with frontal symptomatologies without evident anatomic abnormalities. Both patient groups present subtle frontal behaviors (e.g., impulsiveness, hypersexuality, substance abuse, disinhibition, and/or pathological gambling). By integrating reward and monitoring systems, decision-making provides a direct link to goal-directed action. A better description of these frontal functions may help to diagnose and treat both disorders.

At a theoretical level, our results highlight the role of monitoring systems and their relevance in decision and reward processing [3], [4], [6], [7], [57]. Learning processes triggered by feedback and by choice relevance (e.g., reward magnitude) constitutes an important step in characterizing decision-making. The role of the cingulate cortex in selection and monitoring, along with that of the amygdala and basal ganglia in reward systems, should be affected in patients with ADHD and those with BD.

## References

[1] Bechara A, Van Der Linden M (2005). Decision-making and impulse control after frontal lobe injuries.. Curr Opin Neurol.

[2] Brand M, Labudda K, Markowitsch HJ (2006). Neuropsychological correlates of decision-making in ambiguous and risky situations.. Neural Netw.

[3] Gleichgerrcht E, Ibanez A, Roca M, Torralva T, Manes F (2010). Decision-making cognition in neurodegenerative diseases.. Nat Rev Neurol.

[4] Kable JW, Glimcher PW (2009). The neurobiology of decision: consensus and controversy.. Neuron.

[5] Glimcher PW, Rustichini A (2004). Neuroeconomics: the consilience of brain and decision.. Science.

[6] Krain AL, Wilson AM, Arbuckle R, Castellanos FX, Milham MP (2006). Distinct neural mechanisms of risk and ambiguity: a meta-analysis of decision-making.. Neuroimage.

[7] Rangel A (2008). Consciousness meets neuroeconomics: what is the value of stimulus awareness in decision making?. Neuron.

[8] Chang KD (2010). Course and impact of bipolar disorder in young patients.. J Clin Psychiatry.

[9] Klassen LJ, Katzman MA, Chokka P (2010). Adult ADHD and its comorbidities, with a focus on bipolar disorder.. J Affect Disord.

[10] Wingo AP, Ghaemi SN (2007). A systematic review of rates and diagnostic validity of comorbid adult attention-deficit/hyperactivity disorder and bipolar disorder.. J Clin Psychiatry.

[11] Ernst M, Kimes AS, London ED, Matochik JA, Eldreth D (2003). Neural substrates of decision making in adults with attention deficit hyperactivity disorder.. Am J Psychiatry.

[12] Luman M, Sergeant JA, Knol DL, Oosterlaan J (2010). Impaired decision making in oppositional defiant disorder related to altered psychophysiological responses to reinforcement.. Biol Psychiatry.

[13] Mantyla T, Still J, Gullberg S, Del Missier F (2012). Decision making in adults with ADHD.. J Atten Disord.

[14] Christodoulou T, Lewis M, Ploubidis GB, Frangou S (2006). The relationship of impulsivity to response inhibition and decision-making in remitted patients with bipolar disorder.. Eur Psychiatry.

[15] Jollant F, Guillaume S, Jaussent I, Bellivier F, Leboyer M (2007). Psychiatric diagnoses and personality traits associated with disadvantageous decision-making.. Eur Psychiatry.

[16] Bechara A, Damasio AR, Damasio H, Anderson SW (1994). Insensitivity to future consequences following damage to human prefrontal cortex.. Cognition.

[17] Fernandez-Duque D (2007). Actor/observer asymmetry in risky decision making.. Judgment and Decision Making.

[18] Gehring WJ, Willoughby AR (2002). The medial frontal cortex and the rapid processing of monetary gains and losses.. Science.

[19] Yeung N, Sanfey AG (2004). Independent coding of reward magnitude and valence in the human brain.. J Neurosci.

[20] Montgomery SA, Asberg M (1979). A new depression scale designed to be sensitive to change.. Br J Psychiatry.

[21] Young RC, Biggs JT, Ziegler VE, Meyer DA (1978). A rating scale for mania: reliability, validity and sensitivity.. Br J Psychiatry.

[22] Volkow ND, Wang GJ, Fowler JS, Telang F, Maynard L (2004). Evidence that methylphenidate enhances the saliency of a mathematical task by increasing dopamine in the human brain.. Am J Psychiatry.

[23] Beck A, Brown G, Steer R (1996). Manual for the Beck Depression Inventory-II..

[24] Barratt ES, White R (1969). Impulsiveness and anxiety related to medical students' performance and attitudes.. J Med Educ.

[25] Spielberg C, Gorsuch R, Lushene R (1970). Manual for the Stait-Trait Anxiety Inventory..

[26] Barkley R, Murphy C (1998). Attention-deficit hyperactivity disorder: a clinical workbook..

[27] Torralva T, Roca M, Gleichgerrcht E, Lopez P, Manes F (2009). INECO Frontal Screening (IFS): a brief, sensitive, and specific tool to assess executive functions in dementia.. J Int Neuropsychol Soc.

[28] Weschler D (1997). Wechsler Adult Intelligent Scale III..

[29] Rey A (1941). Psychological examination of a case of posttraumatic encephalopathy.. Archives of Psychologie.

[30] Partington J, Leiter R (1949). Partington's pathway test.. The Psychological Center Bulletin.

[31] Slovic P (1966). Risk-taking in children: Age and sex differences.. Child Development.

[32] Huang MX, Mosher JC, Leahy RM (1999). A sensor-weighted overlapping-sphere head model and exhaustive head model comparison for MEG.. Phys Med Biol.

[33] Baillet S, Garnero L, Marin G, Hugonin JP (1999). Combined MEG and EEG source imaging by minimization of mutual information.. IEEE Trans Biomed Eng.

[34] Tzourio-Mazoyer N, Landeau B, Papathanassiou D, Crivello F, Etard O (2002). Automated anatomical labeling of activations in SPM using a macroscopic anatomical parcellation of the MNI MRI single-subject brain.. Neuroimage.

[35] San Martin R, Manes F, Hurtado E, Isla P, Ibanez A (2010). Size and probability of rewards modulate the feedback error-related negativity associated with wins but not losses in a monetarily rewarded gambling task.. Neuroimage.

[36] Nieuwenhuis S, Holroyd CB, Mol N, Coles MG (2004). Reinforcement-related brain potentials from medial frontal cortex: origins and functional significance.. Neurosci Biobehav Rev.

[37] Chandler RA, Wakeley J, Goodwin GM, Rogers RD (2009). Altered risk-aversion and risk-seeking behavior in bipolar disorder.. Biol Psychiatry.

[38] Fujiwara J, Tobler PN, Taira M, Iijima T, Tsutsui K (2009). Segregated and integrated coding of reward and punishment in the cingulate cortex.. J Neurophysiol.

[39] Hayden BY, Nair AC, McCoy AN, Platt ML (2008). Posterior cingulate cortex mediates outcome-contingent allocation of behavior.. Neuron.

[40] Weber BJ, Huettel SA (2008). The neural substrates of probabilistic and intertemporal decision making.. Brain Res.

[41] Li X, Lu ZL, D'Argembeau A, Ng M, Bechara A (2010). The Iowa Gambling Task in fMRI images.. Hum Brain Mapp.

[42] Peters J, Buchel C (2009). Overlapping and distinct neural systems code for subjective value during intertemporal and risky decision making.. J Neurosci.

[43] Malloy-Diniz L, Fuentes D, Leite WB, Correa H, Bechara A (2007). Impulsive behavior in adults with attention deficit/hyperactivity disorder: characterization of attentional, motor and cognitive impulsiveness.. J Int Neuropsychol Soc.

[44] Crone EA, Jennings JR, van der Molen MW (2003). Sensitivity to interference and response contingencies in attention-deficit/hyperactivity disorder.. J Child Psychol Psychiatry.

[45] Taylor CT, Hirshfeld-Becker DR, Ostacher MJ, Chow CW, LeBeau RT (2008). Anxiety is associated with impulsivity in bipolar disorder.. J Anxiety Disord.

[46] Dunn BD, Dalgleish T, Lawrence AD (2006). The somatic marker hypothesis: a critical evaluation.. Neurosci Biobehav Rev.

[47] Jameson TL, Hinson JM, Whitney P (2004). Components of working memory and somatic markers in decision making.. Psychon Bull Rev.

[48] Gleichgerrcht E, Torralva T, Roca M, Pose M, Manes F (2011). The role of social cognition in moral judgment in frontotemporal dementia.. Soc Neurosci.

[49] Lee D (2008). Game theory and neural basis of social decision making.. Nat Neurosci.

[50] Martino DJ, Strejilevich SA, Torralva T, Manes F (2010). Decision making in euthymic bipolar I and bipolar II disorders..

[51] Clark L, Iversen SD, Goodwin GM (2002). Sustained attention deficit in bipolar disorder.. Br J Psychiatry.

[52] Rubinsztein JS, Michael A, Paykel ES, Sahakian BJ (2000). Cognitive impairment in remission in bipolar affective disorder.. Psychol Med.

[53] Yechiam E, Hayden EP, Bodkins M, O'Donnell BF, Hetrick WP (2008). Decision making in bipolar disorder: a cognitive modeling approach.. Psychiatry Res.

[54] Buelow MT, Suhr JA (2009). Construct validity of the Iowa Gambling Task.. Neuropsychol Rev.

[55] Roiser J, Farmer A, Lam D, Burke A, O'Neill N (2009). The effect of positive mood induction on emotional processing in euthymic individuals with bipolar disorder and controls.. Psychol Med.

[56] Wilbertz G, Tebartz van Elst L, Delgado MR, Maier S, Feige B (2011). Orbitofrontal reward sensitivity and impulsivity in adult attention deficit hyperactivity disorder.. Neuroimage.

[57] Rushworth MF, Behrens TE, Rudebeck PH, Walton ME (2007). Contrasting roles for cingulate and orbitofrontal cortex in decisions and social behaviour.. Trends Cogn Sci.

[58] Biederman J, Makris N, Valera EM, Monuteaux MC, Goldstein JM (2008). Towards further understanding of the co-morbidity between attention deficit hyperactivity disorder and bipolar disorder: a MRI study of brain volumes.. Psychol Med.

[59] Dickstein SG, Bannon K, Castellanos FX, Milham MP (2006). The neural correlates of attention deficit hyperactivity disorder: an ALE meta-analysis.. J Child Psychol Psychiatry.

[60] Fountoulakis KN, Giannakopoulos P, Kovari E, Bouras C (2008). Assessing the role of cingulate cortex in bipolar disorder: neuropathological, structural and functional imaging data.. Brain Res Rev.

[61] Benedetti F, Bernasconi A, Blasi V, Cadioli M, Colombo C (2007). Neural and genetic correlates of antidepressant response to sleep deprivation: a functional magnetic resonance imaging study of moral valence decision in bipolar depression.. Arch Gen Psychiatry.

[62] Torralva T, Gleichgerrcht E, Torrente F, Roca M, Strejilevich SA (2011). Neuropsychological functioning in adult bipolar disorder and ADHD patients: a comparative study.. Psychiatry Res.

[63] Brooks JO, 3rd, Wang PW, Strong C, Sachs N, Hoblyn JC (2006). Preliminary evidence of differential relations between prefrontal cortex metabolism and sustained attention in depressed adults with bipolar disorder and healthy controls.. Bipolar Disord.

[64] Kaladjian A, Jeanningros R, Azorin JM, Nazarian B, Roth M (2009). Reduced brain activation in euthymic bipolar patients during response inhibition: an event-related fMRI study.. Psychiatry Res.

[65] Murphy FC, Sahakian BJ, Rubinsztein JS, Michael A, Rogers RD (1999). Emotional bias and inhibitory control processes in mania and depression.. Psychol Med.

[66] Schecklmann M, Ehlis AC, Plichta MM, Romanos J, Heine M (2008). Diminished prefrontal oxygenation with normal and above-average verbal fluency performance in adult ADHD.. J Psychiatr Res.

[67] Manes F, Sahakian B, Clark L, Rogers R, Antoun N (2002). Decision-making processes following damage to the prefrontal cortex.. Brain.

[68] Clark L, Manes F, Antoun N, Sahakian BJ, Robbins TW (2003). The contributions of lesion laterality and lesion volume to decision-making impairment following frontal lobe damage.. Neuropsychologia.

[69] Clark L, Manes F (2004). Social and emotional decision-making following frontal lobe injury.. Neurocase.

[70] Manes FF, Torralva T, Roca M, Gleichgerrcht E, Bekinschtein TA (2010). Frontotemporal dementia presenting as pathological gambling.. Nat Rev Neurol.

[71] Torralva T, Kipps CM, Hodges JR, Clark L, Bekinschtein T (2007). The relationship between affective decision-making and theory of mind in the frontal variant of fronto-temporal dementia.. Neuropsychologia.

